# The utility of self-expanding metal stents in benign biliary strictures- a retrospective case series

**DOI:** 10.1186/s12876-023-02998-8

**Published:** 2023-10-21

**Authors:** Katlin Mallette, Jeffrey Hawel, Ahmad Elnahas, Nawar A. Alkhamesi, Christopher M. Schlachta, Ephraim S. Tang

**Affiliations:** 1 CSTAR (Canadian Surgical Technologies & Advanced Robotics), London Health Sciences Centre, and Department of Surgery, Western University, London, Canada; 2 Department of Surgery, Division of General Surgery, Schulich School of Medicine and Dentistry, Western University, University Hospital, London Health Sciences Centre, 339 Windermere Road, London, ON N6A 5A5 Canada

**Keywords:** Biliary stricture, Self expanding metal stents, Liver transplant, Plastic stent

## Abstract

**Background:**

Benign biliary strictures can have a significant negative impact on patient quality of life. There are several modalities which can be utilized with the goal of stricture resolution. These techniques include balloon dilatation, placement of multiple plastic stents and more recently, the use of metal stents. The aim of this study was to evaluate the local success of self-expanding metal stents in successfully resolving benign biliary strictures.

**Methods:**

This was a single institution, retrospective case series. Patients included in our study were patients who underwent endoscopic retrograde cholangiopancreatography with placement of self expanding metal stents for benign biliary strictures at our institution between 2016–2022. Patients were excluded for the following: malignant stricture, and inability to successfully place metal stent. Data was evaluated using two-sided t-test with 95% confidence interval.

**Results:**

A total of 31 patients underwent placement of 43 self-expanding metal stents and met inclusion criteria. Mean age of patients was 59 ± 10 years, and were largely male (74.2% vs. 25.8%). Most strictures were anastomotic stricture post liver transplant (87.1%), while the remainder were secondary to chronic pancreatitis (12.9%). Complications of stent placement included cholangitis (18.6%), pancreatitis (2.3%), stent migration (20.9%), and inability to retrieve stent (4.7%). There was successful stricture resolution in 73.5% of patients with anastomotic stricture and 33.3% of patients with stricture secondary to pancreatitis. Resolution was more likely if stent duration was > / = 180 days (73.3% vs. 44.4%, *p* < 0.05). There was no demonstrated added benefit when stent duration was > / = 365 days (75% vs. 60.9%, *p* = 0.64).

**Conclusions:**

This study demonstrates that self expanding metal stents are a safe and effective treatment for benign biliary strictures, with outcomes comparable to plastic stents with fewer interventions. This study indicates that the optimal duration to allow for stricture resolution is 180–365 days.

## Background

Benign biliary strictures (BBS) are common issue confronted by surgeons and endoscopists. Many of these patients present with obstructive jaundice which requires intervention [1, 2]. There are many underlying causes of development of BBS and they can often be difficult to differentiate from malignant biliary strictures [1]. The most common causes of BBS are cholecystectomy complications, chronic pancreatitis, and anastomotic strictures related to orthotopic liver transplantation [2–6]. Anastomotic strictures post transplant place the transplant at significant risk of failure and need for re-transplantation [5–7].

Strictures related to chronic pancreatitis and liver transplant are often difficult to treat, with high rates of recurrence. Many modalities have been used in their treatment over the years, with endoscopic management being the mainstay [1, 2, 8–11]. Initially, treatment with sphincterotomy and balloon dilatation alone was utilized [7, 9, 12]. Unfortunately, long term outcomes with this procedure were quite poor, with only 56% long term stricture resolution [9]. Insertion of multiple plastic biliary stents has since become the mainstay and gold standard treatment of these strictures [8, 9, 12, 13]. The success rate of multiple plastic stents has been demonstrated to be as high as 90% in post-operative patients. More recently, the introduction of fully covered self expanding metal stents (SEMS) has shown promise in the area of BBS [14]. However, the uptake has been slow and many guidelines continue to recommend placement of multiple plastic stents as primary treatment [15–17].

The aim of our study was to evaluate the local success of SEMS in resolving benign biliary strictures, and compare outcomes with the use of plastic stents.

## Methods

The STROBE checklist was utilized to ensure completeness of reporting. A single institution, retrospective case series was carried out between January 2016 and July 2022. Patients eligible to be included in the study were those undergoing placement of SEMS for benign biliary stricture. These patients were identified using a hospital database consisting of all patients undergoing placement of SEMS for any reason. All patients over the age of 18 who underwent SEMS placement at London Health Sciences Centre (University Hospital and Victoria Hospital) in London, Canada for benign biliary stricture were included in the study. Patients were excluded if they underwent stent placement for malignant indications or if stent placement was not possible at endoscopic retrograde cholangiopancreatography (ERCP). All methods were carried out in accordance with relevant guidelines and regulations, and informed consent was obtained from all patients prior to their procedure.

At our institution post-transplant patients presenting with a benign biliary stricture are managed by a surgical endoscopist who also performs liver transplant. At initial presentation these patients are managed with a single plastic stent as first line. The plastic stent is then exchanged for a self-expanding metal stent once the obstruction is relieved. The metal stent is then left in situ for 1 year if possible. If there is stricture recurrence or non-response, consideration is given to repeat stenting versus conversion to a roux limb. For patients presenting with benign biliary strictures secondary to chronic pancreatitis, management is at the discretion of the treating endoscopist.

Electronic patient charts of all patients eligible to be included in the study were reviewed and the required data was extracted and recorded. Patient demographic data collected included: age, biological sex, and presence/absence of anticoagulation. Disease characteristic data included: underlying diagnosis, previous plastic stent, previous placement of SEMS, duration of disease, and sphincterotomy. Procedure related data collected included: duration of stent, type/length of stent placed and number of stents. Peri-procedure data included: rates of stent migration, perforation, pancreatitis, gastrointestinal bleed, operative intervention, stent related admission, need for repeat transplant and resolution of stricture.

Stricture resolution was defined as lack of significant upstream dilatation of biliary tree on cholangiogram and easy passage of balloon catheter at time of ERCP, as well as resolution of hyperbilirubinemia/elevated cholestatic liver enzymes. Stricture recurrence was defined as presence of obstructive pattern on liver enzymes triggering ERCP which demonstrated narrowing of biliary tree with associated upstream biliary dilatation and difficulty passing cholangiogram balloon. All peri-procedure complications were based on standard definitions. Pancreatitis was defined as lipase greater than 3 times the upper limit of normal with associated abdominal pain. Cholangitis was defined by the presence of Charcot’s triad or Reynold’s pentad as documented in the patient’s electronic chart. Gastrointestinal bleed was defined as a drop in hemoglobin from baseline, associated with overt signs of an upper GI bleed, and endoscopic evidence of bleeding. All SEMS were placed with the aid of fluoroscopic guidance to ensure proper placement of the stent across the stricture, and ensure the distal aspect of the stent protruded through the ampulla to allow for easy stent retrieval.

Analysis of the two groups was undertaken using standard statistical methods (mean/percentage), as well as two-sided unpaired t-test with a 95% confidence interval.

## Results

**Fig. 1:**
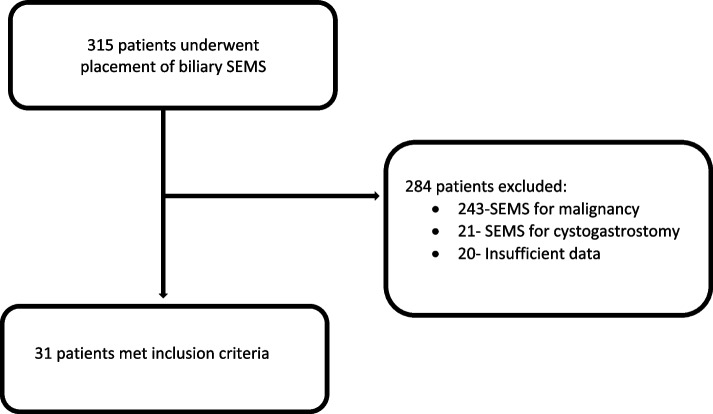
Flow diagram demonstrating patients included in the study and those excluded

A total of 31 patients underwent placement of SEMS between January 2016-July 2022 at our institution and met inclusion criteria (Fig. 1). Most patients required only a single metal stent (range: 1–3) for a total of 43 stents placed among the 31 patients. The baseline characteristics of patients included in the study are outlined in Table 1. Three patients (9.7%) were on anticoagulation at the time of stent placement; two were on factor Xa inhibitors, and one was on an antiplatelet medication. For patients with liver transplant and subsequent anastomotic stricture, the underlying etiology of liver cirrhosis prior to liver transplant and type of anastomosis is indicated in Table 2.

**Table 1:** Baseline characteristics of included patients

**Characteristic**	
**Age**	59 ± 10
**Gender (Male/Female)**	74.2%/25.8% (25/8)
**Diagnosis**	
Anastomotic stricture	87.1% (27)
Chronic pancreatitis	12.9% (4)
**Disease Duration**	
< 1 year	64.5% (20)
1–5 years	22.6% (7)
> 5 years	6.5% (2)
Unknown	6.5% (2)
**Previous Metal Stent**	9.7% (3)
**Previous Plastic Stent** Mean ± stdev	1 ± 2
**History of Sphincterotomy**	100% (31)

**Table 2:** Underlying etiology of liver cirrhosis and anastomotic technique for patients presenting with post liver transplant anastomotic stricture

**Characteristic**	
**Cirrhosis Etiology** n (%)	
Alcohol	10 (37.0%)
Non-Alcoholic Steatohepatitis	4 (14.8%)
Hepatitis B	2 (7.4%)
Hepatitis C	8 (29.6%)
Primary Sclerosing Cholangitis	1 (3.7%)
Budd-Chiari Syndrome	1 (3.7%)
Drug Induced	1 (3.7%)
**Type of Anastomosis** n(%)	
End-to-end choledocho-choledochostomy	26 (96.3%)
End-to-side choledocho-duodenostomy	1 (3.7%)

All patients had placement of only a single SEMS at each necessary intervention, the vast majority of stents were fully coated metals stents (*n* = 42, 97.7%), with only a single uncovered stent used. Further specifics of stents utilized are outlined in Table 3. Mean duration of stent placement was 334 ± 218 days, with a maximum duration of 1096 days, and a minimum duration of 2 days. Patients underwent an average of two endoscopic procedures during the course of the study.

**Table 3:** Specifications of stents utilized

Specification	Number	%
**Stent type**		
Kaffes	20	46.5%
Cook Evolution	10	23.3%
Wallflex	6	14.0%
Unknown	7	16.3%
**Stent Size**		
10mmx6cm	34	79.1%
10mmx8cm	9	20.9%

Overall success rate of stricture resolution was 93.0% (*n* = 40). Rates of stricture resolution were not statistically significantly different between patients with post-transplant anastomotic strictures compared to strictures secondary to chronic pancreatitis (Transplant: *n* = 25, 73.5% vs. CP: *n* = 2, 50.0%; *p* = 0.05), but there was a trend toward better resolution in transplant patients. Stricture resolution occurred more frequently in patients with stent duration of more than 180 days (< 180 days: *n* = 4, 44.4% vs. ≥ 180 days: *n* = 22, 73.3%; *p *< 0.05). This difference was not maintained when evaluating success for stents longer than 365 days (< 365 days: *n* = 14, 60.9% vs. ≥ 365 days: *n* = 12, 75.0%; *p* = 0.64).

Overall, rates of perioperative complications were low. Stent migration was the most common complication (*n* = 9, 20.9%), followed by cholangitis (*n* = 8, 18.6%). A single patient (2.3%) developed post-procedure pancreatitis, while the stent was not able to be retrieved in 1 patient (2.3%). There were no documented cases of perforation or post-ERCP hemorrhage. No patients required operative intervention associated with the intervention. Ten patients (23.3%) required admission related to their stents, of those patients 80% (*n* = 8) for cholangitis, 10% (*n* = 1) for pancreatitis and 10% (*n* = 1) for elevated liver enzymes. Of those patients admitted with cholangitis, 87.5% (*n* = 7) were patients with anastomotic strictures, and 12.5% (*n* = 1) were patients with chronic pancreatitis. Cholangitis occurred within 72 h of ERCP in 37.5% (*n* = 3), due to stent occlusion in 37.5% (*n* = 3), and due to proximal migration in 25% (*n* = 2). Mean time to stent occlusion leading to cholangitis was 240 ± 68 days. Ultimately, 2 patients (7.4%) required repeat transplantation due to persistent, non-resolving biliary stricture.

## Discussion

This study is a retrospective, single institution cohort study evaluating patients with BBS undergoing placement of SEMS, with the aim of evaluating the success of SEMS for stricture resolution. The main findings of the study were that patients required fewer endoscopic procedures than traditional treatment with multiple plastic stents, there were higher stricture resolution rates with SEMS and the optimal duration of SEMS placement was 180–365 days.

The average age of patients included in our study was 59 years, indicating that BBS are prevalent among young individuals. This means that treatment methods for this condition, specifically for the transplant population, need to have very high success rates due to the extreme health burden it places on this patient population but also the healthcare system as a whole. [8, 18]. With the exception of one patient, all other SEMS placed were fully covered stents. Uncovered and partially covered stents are contraindicated in patients with BBS due to the risk of tissue ingrowth and inability to remove the stent [8, 9, 11, 19–21]. The patient who had placement of an uncovered stent in our study had a pancreatic intraductal papillary mucinous neoplasm and the stricture was thought to be malignant at placement.

The majority of patients in our study had an underlying diagnosis of anastomotic stricture secondary to liver transplant, with a smaller number of patients with BBS secondary to chronic pancreatitis. This difference is likely due to our institution being a liver transplant centre. Disease duration was typically less than 1 year in most patients evaluated. This is consistent with the data that demonstrates that most post-transplant anastomotic strictures present within the first year after transplantation [22].

Patients on average underwent two procedures prior to stricture resolution. This is consistent with the literature, where the patients with SEMS typically underwent 2–3 procedures [3, 7, 23]. This is significantly fewer procedures than those with multiple plastic stents, who on average have 3–5 procedures [7, 23]. Consequently, the overall cost of SEMS has been found to be lower than placement of multiple plastic stents [3, 4, 24]. Stent duration in this study was on average 344 days, which is longer than has been previously documented in multiple studies. Many studies evaluating SEMS have utilized removal of the stent at 3 months with good success [3, 7, 11, 25, 26]. On the other hand, the recommended treatment protocol for multiple plastic stents is stent exchange every 3 months for 1–2 years [1, 2, 7, 8, 27].

SEMS demonstrated high rates of stricture resolution in our study, with 93% of patients achieving stricture resolution, with patients with chronic pancreatitis demonstrating lower rates. This is similar to that seen in other previous studies, including multiple systematic reviews and meta-analyses demonstrating good success of SEMS and similar rates in comparison to multiple plastic stents [1, 9, 21, 24, 28–30]. Rates of stricture resolution in patients with placement of multiple plastic stents has been shown to be similar. A 2009 systematic review demonstrated success rates of 94.3% in patients with BBS stricture treated with multiple plastic stents [20].

Rates of stricture resolution were not statistically different between those with post-transplant anastomotic strictures compared to those secondary to chronic pancreatitis, this is likely due to low patient numbers in both groups. However, it has been demonstrated previously that rates of stricture resolution are higher in patients’ post-liver transplant compared to those with chronic pancreatitis [5, 6, 16, 31, 32]. In patients with anastomotic strictures post orthotopic liver transplants, the use of fully covered SEMS has been shown to have stricture resolution rates as high as 100%, with low rates of complications (6.5%) [22]. Success in patients with chronic pancreatitis is typically much lower. Two recent multicenter, randomized control trials comparing multiple plastic stents with SEMS have shown similar rates between the two techniques, with modest stricture resolution [31, 32]. One RCT published in 2021 assessing 80 patients after placement of fully covered SEMS for symptomatic chronic pancreatitis associated benign biliary strictures, demonstrated resolution in 75.8% of patients at two years [31].

Rates of recurrence were not evaluated in our study, but have been shown to be similar to those of plastic stents at 7–20% [24, 28–30]. In our study, stent duration of 180–365 days appeared to be optimal for stricture resolution. This appears to be contrary to previous studies, which have only left SEMS in place for 3–4 months, despite evidence to suggest that SEMS patency remains up to ~ 1 year after placement [11]. Our study suggests that leaving SEMS in situ for longer than 3 months may be more effective at stricture resolution.

The overall rate of complications seen in this study are comparable to that seen in the literature for SEMS. Systematic reviews have shown that SEMS have overall higher rates of complications that multiple plastic stents, with rates found in the literature of 20.3%. The most crucial issue with SEMS is the rate of stent migration. In our study, the rate was 20.9%, and 25% of patients admitted with cholangitis secondary to stent migration. This is concordant with that shown in the literature of 16–40% rates of stent migration [8, 14, 28, 30, 33]. Many companies have attempted to mitigate this risk with the use of a variety of techniques including anti-migration flares [14, 22, 28]. Despite these advances, the rate of migration remains significantly higher than plastic stents of 0–8.6%. Further, the vast majority of migrated plastic stents will pass spontaneously, however, there is some concern that migrated SEMS occasionally require endoscopic removal [8]. It has been postulated that post-ERCP pancreatitis is substantially more frequent in SEMS placement [24, 33]. However, the rate in our study was only 2.3% which is similar to the baseline risk of post-ERCP pancreatitis [34]. Another concern with placement of SEMS is the difficulty with stent retrieval. There was only one patient in whom stent could not be retrieved. At the time of the completion of this study, this patient remained well with the stent in situ for 1131 days, with no evidence of cholangitis, with the stent in situ. They continue to be closely monitored. This risk is substantially higher in uncovered or partially covered SEMS due to tissue ingrowth and is mitigated by using fully covered stents [4, 8, 11, 19, 20]. Neither of these patients suffered observed adverse events secondary to inability to remove stent during the study period.

Three liver transplant patients were admitted within 72 h of ERCP with cholangitis despite receiving a single dose of peri-procedure antibiotics. Guidelines remain unclear on the need for peri-procedural antibiotics in this patient population when adequate drainage is achieved [35, 36]. Further research needs to be done to determine which patients may benefit most from single dose versus extended antibiotic prophylaxis. Stent occlusion led to admission for cholangitis in three patients with stent duration ranging from 163 to 291 days. Given the optimal stent duration of 180–365 days noted in this study, close follow up and monitoring of patients with SEMS is critical to allow for early intervention in this patient population. It has been postulated that patients with SEMS placement across the ampulla may have higher rates of cholangitis secondary to reflux of duodenal effluent with bacteria. However, a recent systematic review published in the Scandinavian Journal of Gastroenterology demonstrated no increased rates of cholangitis in patients with stents placed across the ampulla [37]. Therefore, it is important to monitor for cholangitis in all patients with strictures, but stent placement above the ampulla is not instrumental.

This study has some limitations, including the small sample size and potential selection bias. Furthermore, given the retrospective nature of this study, procedures not occurring at our institution may not have been captured in our dataset. Currently, at our institution the protocol for placement of SEMS for BBS is to leave the stent in situ for 6–12 months, however, due to limitations on endoscopy service and patient compliance, this is not always the case. This leads to large variation in stent duration.

## Conclusions

In conclusion, placement of fully covered SEMS appears to be a relatively safe and effective treatment in patients with BBS, specifically those in patients with post liver transplant anastomotic strictures. The optimal duration of stent placement is between 180–365 days, and ultimately leads to fewer endoscopic procedures. However, patients need to be monitored very closely for stent migration which can lead to significant challenges in management of these patients, as well as monitoring for the development of cholangitis. Further research needs to be done to develop protocols for treatment of recurrent strictures and develop more reliable fully covered SEMS with low risk of migration.

## Data Availability

The datasets used and analysed during the current study are available from the corresponding author on reasonable request.

## Abbreviations

BBS: Benign biliary strictures
SEMS: Self expanding metal stents
ERCP: Endoscopic retrograde cholangiopancreatography
